# Cholinergic Plasticity of Oscillating Neuronal Assemblies in Mouse Hippocampal Slices

**DOI:** 10.1371/journal.pone.0080718

**Published:** 2013-11-18

**Authors:** Maura M. Zylla, Xiaomin Zhang, Susanne Reichinnek, Andreas Draguhn, Martin Both

**Affiliations:** Institute of Physiology and Pathophysiology, University Heidelberg, Heidelberg, Germany; Georgia State University, United States of America

## Abstract

The mammalian hippocampus expresses several types of network oscillations which entrain neurons into transiently stable assemblies. These groups of co-active neurons are believed to support the formation, consolidation and recall of context-dependent memories. Formation of new assemblies occurs during theta- and gamma-oscillations under conditions of high cholinergic activity. Memory consolidation is linked to sharp wave-ripple oscillations (SPW-R) during decreased cholinergic tone. We hypothesized that increased cholinergic tone supports plastic changes of assemblies while low cholinergic tone favors their stability. Coherent spatiotemporal network patterns were measured during SPW-R activity in mouse hippocampal slices. We compared neuronal activity within the oscillating assemblies before and after a transient phase of carbachol-induced gamma oscillations. Single units maintained their coupling to SPW-R throughout the experiment and could be re-identified after the transient phase of gamma oscillations. However, the frequency of SPW-R-related unit firing was enhanced after muscarinic stimulation. At the network level, these changes resulted in altered patterns of extracellularly recorded SPW-R waveforms. In contrast, recording of ongoing SPW-R activity without intermittent cholinergic stimulation revealed remarkably stable repetitive activation of assemblies. These results show that activation of cholinergic receptors induces plasticity at the level of oscillating hippocampal assemblies, in line with the different role of gamma- and SPW-R network activity for memory formation and –consolidation, respectively.

## Introduction

The mammalian hippocampus plays a crucial role in spatial and declarative memory formation. The underlying neuronal mechanisms are likely to involve activity-dependent changes in coupling of local neurons, thereby forming transiently stable assemblies [1], [2], [3]. According to this concept of plasticity, environmental cues result in co-activation of defined neurons, encoding for segments of episodic memory, which stabilize their connections [4], [5], [6]. Subsequently, similar contexts are sufficient to activate the full assembly of previously linked neurons, even upon partial cue presentation [7], [8] or during sleep [9], [10]. Thus, hippocampal networks must keep a balance between experience-dependent plasticity of neuronal connections to allow the encoding of novel information and, at the same time, stability of the resulting assemblies for reliable storage and readout.

Recent evidence indicates that formation and consolidation of hippocampal assemblies occur during different functional network states [2], [11], [12]. Active exploratory behavior goes along with hippocampal theta rhythms (5–10 Hz) which are superimposed by gamma oscillations (30–100 Hz) [13], [14], [15]. During these activity patterns, afferent fibers from septal nuclei release acetylcholine [16]. Acetylcholine induces persistent spiking in pyramidal cells and supports short- and long-term potentiation of synaptic connections. Various experiments have shown that cholinergic modulation lowers the threshold for induction of long-term potentiation (LTP) at different synapses including the Schaffer collaterals between CA3 and CA1 [17], [18], [19]. Moreover, activation of muscarinic receptors enhances synchronous firing of CA1 pyramidal neurons [20] and induces gamma oscillations in hippocampal networks [21]. Therefore, high acetylcholine levels during waking episodes provide the hippocampus with favorable conditions for encoding new information while reducing interference from previously established patterns. Cholinergic activity may, thereby, contribute to assembly formation [4], [22].

Conversely, during episodes of inactivity or slow-wave sleep (SWS), a reduction in acetylcholine level releases existing connections from inhibition and enables transmission of established spatiotemporal patterns to the neocortex [23], [24] This state may, thus, support recall and consolidation of memories. The accompanying network state has been characterized as large irregular activity [13] involving propagating sharp waves (SPW) which are superimposed by trains of fast (∼200 Hz) network oscillations, called ripples. Sharp waves are generated in CA3 and travel along the hippocampal output loop towards the entorhinal cortex [25], [26]. Based on the observed re-play of previously acquired neuronal discharge patterns [9], [27] sharp wave-ripple complexes (SPW-R) have been proposed to mediate memory consolidation [11]. Consistently, disruption of SPW-R during SWS [28], [29] or during waking states [30] impairs spatial memory performance in rodents.

At the network level, these findings suggest that cholinergically-driven gamma oscillations support activity-dependent plasticity of assemblies whereas SPW-R stabilize pre-existing groups of co-active neurons. We tested this hypothesis using an *in vitro* model of hippocampal network oscillations in mouse hippocampal slices [31]. Spontaneous SPW-R-activity was interrupted by a transient episode of muscarinic receptor activation which reliably induced gamma oscillations. This intermittent neuromodulatory input specifically enhanced network-entrained unit activity during subsequent SPW-R. In addition, waveforms of field-SPW-R were altered, indicating changes of the underlying assemblies. Control slices with ongoing SPW-R activity showed mostly unchanged assemblies. Our findings therefore demonstrate plasticity at level of neuronal assemblies which is driven by neuromodulatory influences on network oscillations.

## Methods

The study was conducted in accordance with German animal protection law. All procedures were approved by the state government of Baden-Württemberg (T – 08/10).

### Slice preparation

Experiments were performed on young adult male C57BL/6 mice (4–12 weeks). Following deep anesthesia mice were decapitated and the brain was removed. Afterwards, the brain was transferred into cooled artificial CSF (ACSF; 1–4°C) of the following composition (in mM): 124 NaCl, 3.0 KCl, 1.8 MgSO_4_, 1.6 CaCl_2_, 10 Glucose, 1.25 NaH_2_PO_4_, and 26 NaHCO_3_. The ACSF was saturated with carbogen gas (95% O_2_, 5% CO_2_) and a physiological pH of 7.4 was maintained. Frontal brain structures and the cerebellum were truncated and horizontal slices of 450 µm were prepared using a Leica Vibratome (VT1000 S). After transferring the slices to a Haas-type interface recording chamber they were maintained at 32±0.5°C at the interface between ACSF and warm, moist carbogen gas. Two hours of recovery under these conditions preceded the start of measurements.

### Recordings

Local field potentials and unit discharges were recorded with custom-made tetrodes which were made of four twisted 12.5 µm-diameter tungsten wires (California Fine Wire, Grover Beach, USA). Tetrodes were carefully lowered onto the principal cell layers of CA1, CA3 and then slightly inserted into the surface of the slice. Each tetrode channel was connected to a DPA-2FX amplifier (npi electronics). Signals were amplified 100×, low-pass filtered at 10 kHz, high-pass filtered at 0.3 Hz, and digitized at 20 kHz for off-line analysis (1401 interface and Spike-2 data acquisition program; CED).

### Pharmacology

Gamma oscillations were induced by bath-application of carbachol (CCh, 20 µM, Sigma). Muscarinic receptor activation was blocked by bath-application of atropine (1 µM, Sigma) to re-establish SPW-Rs. Solutions containing drugs were prepared freshly from 1000-fold concentrated stock solutions in ACSF.

### Experimental protocols

In order to test the effect of transient gamma oscillations under cholinergic tone on network activity during SPW-R, we recorded the slices under baseline conditions (spontaneous SPW-R-activity) for 40 min. Subsequently, carbachol (CCh) was applied. Gamma oscillations developed within ∼30–45 min and were analyzed at 60–105 min. Subsequently, atropine was washed in for one hour followed by a second SPW-R recording episode of at least 40 min. These experiments are called *CCh* throughout the manuscript.

Slices that displayed epileptiform potentials in the presence of carbachol, e.g. high-amplitude spikes or polymorphic repetitive discharges, were excluded from further data acquisition.

To analyze network and unit properties during ongoing SPW-R states, we recorded for 40 min (baseline episode) followed by an additional drug-free interval of 3 hours (corresponding to the time of carbachol application, gamma recording time and atropine application, see below). Subsequently, we recorded SPW-R activity for a second episode of at least 40 min. This group of experiments additionally served as a control for longterm-variations of activity within the slices and is called *SPW-R* throughout the manuscript.

Controls for effects of atropine (named *atropine* in the manuscript) were performed by 40 min of baseline recording followed by a 1-hour wash-in of atropine and subsequent recording for at least 40 min.

To detect changes in synaptic transmission after an intermediate episode of gamma oscillations, we electrically stimulated the Schaffer collaterals with bipolar platinum/iridium wire electrodes (75 µm tip distance, 100 kOhm at 1 kHz, Science Products Trading, Hofheim, Germany) in a subset of slices. Stimulation strength was adjusted to obtain 60% of the maximal population spike amplitude and 100 µs long pulses were delivered every 60 s.

In order to ensure stability of slice quality only experiments which did not show more than 10% variation in SPW-amplitude and –frequency during the analyzed timespan of baseline recordings were chosen for data evaluation.

### Data Analysis

Recording was done with Spike-2 (CED) and analyzed off-line after conversion of data files into Matlab (The MathWorks, Natick, MA). The last 1000 s before induction of gamma oscillation and the first 1000 s after stable re-establishment of SPW-R by atropine application were chosen for data analysis. The corresponding time windows were chosen for experiments without intermittent carbachol application.

For sharp wave detection, raw data was first down-sampled to 1000 Hz to minimize computational load. Then, the down-sampled data was low-pass filtered (<60 Hz) and events were detected as local maxima exceeding 0.08 mV. This value corresponded to ∼4 standard deviations (SD) of event-free baseline noise [32]. Ripples were detected by down-sampling raw data to 2.5 kHz, band-pass filtering (140–300 Hz) and detecting local minima with amplitudes larger than 3 SD of event-free baseline noise. This analysis was done on one of the tetrode channels. Units were detected on all four tetrode channels from high pass-filtered (>500 Hz) raw data. Single events were identified by setting a negative threshold at 4.5 SD of event-free baseline noise. Subsequently, 1 ms of high-pass filtered data was extracted around each detected event on all four tetrode channels and up-sampled to 100 kHz with a Nyquist algorithm [33]. This method renders a mathematically correct reconstruction of the original spike waveform at higher time resolution than the original sampling rate. As a prerequisite, the highest frequency in the recorded data must be lower than half the original sampling rate (Nyquist theorem). This was ensured by our analog filter setting (10 kHz). Thus, the up-sampled spike waveforms provide more detailed information for the next step of analysis, while keeping needs for hard disk and computer memory space minimal. Subsequently, data were analyzed by principal component analysis (PCA) and clustered using the first three principal components of each tetrode channel with the open source program KlustaKwik [34]. Final clustering of single units was performed by visual inspection with the open source program Klusters [35]. Criteria for the definition of single units were a clear separation from other events, stable waveforms and a clear refractory period in the auto-correlogram. Coupling precision of unit firing to field ripples was computed by assigning to each event a phase within one ripple cycle [36]. Ripple cycles were described as circular data of 360 degrees with ripple troughs set to zero degree. From these phases, the mean preferred firing angle and the precision was calculated. Precision is described by the length of the respective mean vector which corresponds to the reciprocal of the jitter. These parameters were determined for units with at least 10 SPW-R-associated action potentials which allowed for reliable calculation.

Unit-to-ripple coupling can only be adequately analyzed if the field ripple can be distinguished from the units. In CA3 field ripples are less pronounced that in CA1. Therefore, only those slices were taken for CA3 unit-to-ripple coupling if a distinct fast component in CA3 frequency spectrograms was detectable.

Changes in SPW-R-related firing of single units were assessed by computing a plasticity coefficient from the number of SPW-R-related discharges before and after a transient episode of gamma oscillations: 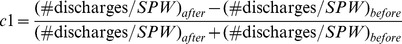



Changes in firing rates outside SPW-R were computed in a similar way from action potential occurring outside SPW-R: 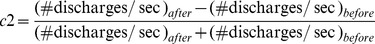



Positive values denote an increase, negative values a decrease in activity. A plasticity coefficient of -1 results from a unit which fires during the initial episode but is lost after the transient gamma oscillation. Conversely, a value of +1 would indicate a ‘new’ unit which was not present in the control recording prior to gamma. Units with unchanged activity before and after the gamma episode result in a coefficient of 0. Data without intermitted gamma oscillations were processed in an analogous manner.

### Classification of SPW-R waveforms to investigate underlying assemblies

We have previously shown that different SPW-R waveform patterns reflect activation of different neuronal assemblies [37]. In order to measure stability and plasticity of SPW-R, we compared the different waveforms throughout the experimental time of more than 4 hours. Processing of SPW-R waveforms followed the published protocol [37] In short, local field potentials of SPW-R were band-pass filtered between 10–1,000 Hz, down-sampled to 5 kHz and single SPW-R events were cut out from 33 ms before the peak to 67 ms after the peak constituting 500 time points. Individual events were subjected to principal component analysis and described by the first ten components in the resulting parameter space. We then categorized SPW-R waveforms by their best-matching units on self-organizing maps (SOM, implementation of the Matlab code by Laboratory of Computer and Information Science, Helsinki University of Technology; http://www.cis.hut.fi/projects/somtoolbox; parameters set to “training long” and “lattice rectangle”). When this procedure is applied to the entire data set, it is possible to construct a ‘hit map’ to visualize the classification: the rate of a particular map unit being the ‘best matching map unit’ (BMMU) for an individual SPW-R is displayed by a color code. To test the time evolution of SPW-R waveform composition, we constructed a reference map from the initial 600 s of the recording. We then constructed partial data maps of 100 s segments over the full duration of the experiment, i.e. for the 1000 s of the baseline episode and for the 2000 s of the post-gamma or post-ongoing SPW-R episode (‘post-state’ episode), respectively. Distance between each of these partial data maps and the reference map was calculated as Euclidian distance of all units from one map and the respective best matching map units from the other map (independently performed for both directions between reference and partial maps). The mean of all distances calculated for a pair of maps is called the mean distance throughout the manuscript. To average different experiments, mean distances of each experiment were normalized to the initial 1000 s. Recordings were analyzed as non-normalized data as well as after normalizing to median SPW-R amplitude in order to control for the effects of mere amplitude increases after gamma. In this case we normalized the 1000 baseline episode and the 2000 s ‘post-state’ episode to its respective median SPW-R amplitude before performing the principal component analysis.

### Statistics

Quantitative results are described as mean values ±SEM if normally distributed. In all other cases we specify the median value and the 25^th^ and 75^th^ percentiles. Statistical significance of group differences was calculated by Student's t-test or ANOVA for normal distributions. Data which were not normally distributed were evaluated by Wilcoxon rank sum test or nonparametric ANOVA. Post-hoc analysis was done by Dunn's multiple comparisons test. Tests of plastic changes in single experimental groups were performed by testing against the expected value for constant activity (e.g., a plasticity coefficient of 0) using one-sample t-test for Gaussian distributions and Wilcoxon signed rank test for nonparametric distributions. Correlation between parameters was tested by calculating Spearman coefficients and corresponding p-values. Firing phases within oscillation cycles were compared by applying the Watson-Williams test for circular data. Values of p<0.05 were regarded as significant.

## Results

### Recording of sharp waves and intermittent gamma activity *in vitro*

**Figure 1:**
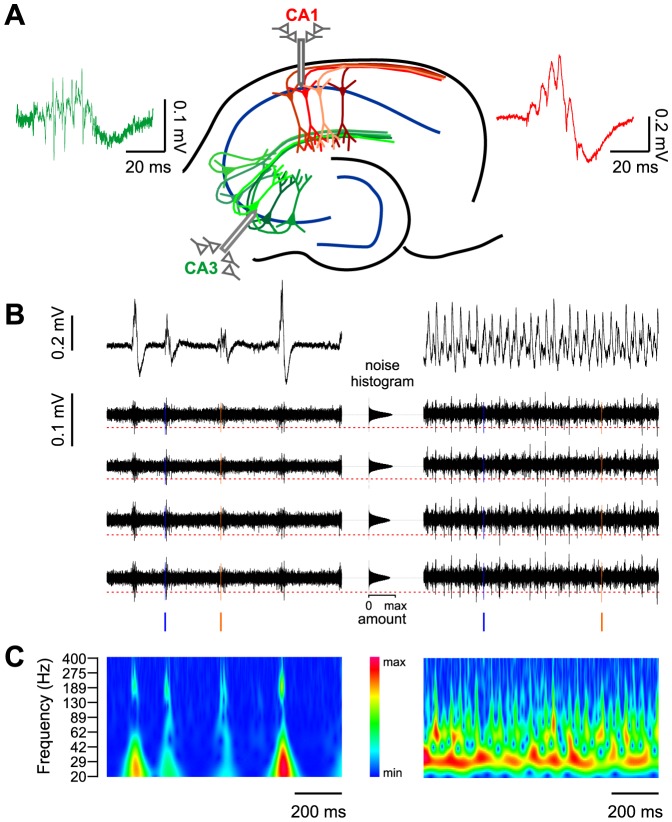
Different network states and corresponding unit activity can be analyzed in the in vitro model. **A**: Schematics of recording conditions. Tetrode recordings are performed in the pyramidal cell layers of CA3 and CA1. During sharp wave-ripple oscillations (SPW-R), field potentials are generated by the recurrent network of CA3 (green) and transmitted via the Schaffer collaterals to selectively activate cell assemblies in CA1 (red). Examples of field potential waveforms are shown in the respective colors. **B**: Example traces of field potentials recorded in CA1 during SPW-R (left panel) and gamma oscillations (right panel). Raw field potential (upper trace) and the high pass filtered (0.5–10 kHz) potential in the four tetrode channels (lower traces). Filtering reveals high-frequency multiunit activity on SPW-R and gamma cycles. Firing of two individual units extracted by waveform analysis is highlighted in orange and blue. Time points of firing relative to the field potential are visualized by vertical ‘ticks’. The middle panel illustrates the detection criterion for unit events: horizontal histograms of all data points show largely normal distribution around zero. Events are detected if they exceed 4.5 times the standard deviation as indicated by the dashed red line. **C**: Wavelet spectrograms of the example traces from B. Sharp waves are superimposed by high-frequency ripple oscillations (∼200 Hz, left panel). The carbachol-induced gamma state consists of a continuous oscillation at ∼30 Hz.

Tetrode recordings from CA1 and CA3 pyramidal cell layers of drug-naïve horizontal mouse hippocampal slices revealed oscillating field potentials and temporally coupled unit discharges (Fig. 1A–C). Spontaneous field potential transients were reminiscent of *in vivo* sharp wave-ripple complexes (SPW-R), as previously described [31] In accordance with their known propagation pattern *in vivo*, SPW-R in CA1 were regularly preceded by high-frequency network bursts in CA3 [32], [38]. In CA1, frequency of SPW-R was 2.85±0.13 Hz (n = 44 slices), and the mean frequency of superimposed ripples was 224.8±3.40 Hz (range 174.1–263.9 Hz).

Upon bath-application of carbachol (20 µM), spontaneous SPW-R activity ceased and was replaced by ongoing oscillations in the gamma frequency band (Fig. 1B and 1C) [21]. These oscillations reached stable frequencies after <60 minutes (mean peak frequencies 37.26±2.63 Hz in CA1, 35.19±3.16 Hz in CA3, n = 15). Thus, recordings in ACSF with and without addition of a muscarinic agonist reveal two different and mutually exclusive patterns of network activity.

We hypothesized that an intermittent episode of muscarinic receptor activation induces plastic changes in the neuronal activity patterns underlying SPW-R. For a systematic comparison, we analyzed two groups of slices: one group (“*CCh*”; n = 15 slices) was exposed to carbachol after recording baseline SPW-R activity. Muscarinic effects were then antagonized by application of atropine (1 µM) in order to return to the SPW-R mode and look for plastic changes. The other group of slices was recorded in a time-matched protocol of ongoing SPW-R without carbachol (“*SPW-R*”, n = 21 slices). We additionally controlled for direct effects of atropine in a third group of slices (“*atropine*”; n = 8 slices).

### Units can reliably be re-identified after an intermittent episode of cholinergically induced gamma oscillations

**Figure 2:**
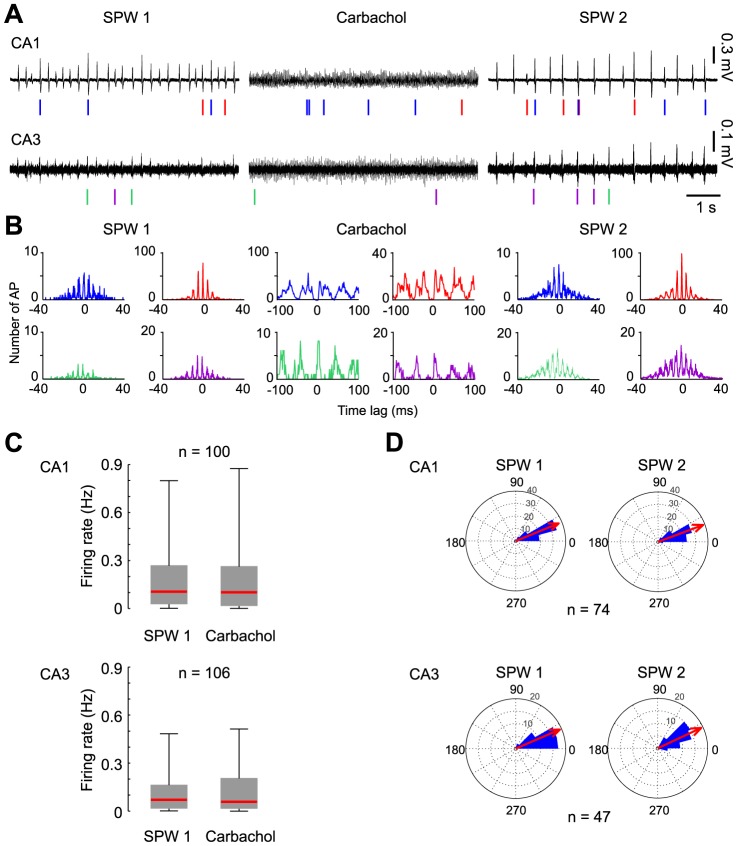
Hippocampal units maintain coupling precision to the local field potential during alternating network states. **A**: Local field potential recordings and single unit activity (symbolized by vertical ticks) in CA1 and CA3 during the three experimental steps of the *CCh* experiments: baseline recording (SPW 1, left panel) followed by carbachol-induced gamma oscillations (Gamma, middle panel) and re-established SPW by wash-in of atropine (SPW 2, right panel). Two representative units are indicated by colored ticks in each region. Note that the individual units could be observed in all three phases of the experiment. **B**: Event cross-correlograms of field-potentials and firing time points of the units depicted in A. During SPW 1 and SPW 2, unit firing is correlated to the ripple-oscillation troughs in CA1 (peak intervals at ∼5 ms, corresponding to ripple cycle length); during the gamma episode they are correlated to the local gamma oscillation troughs (peak intervals at ∼30 ms). **C**: Mean firing rates of units remain constant during gamma oscillations. **D**: Firing phases of units to ripple troughs remain stable after an intermittent episode of gamma oscillations. The ripple cycle is described by a circular scale of 360° and the ripple trough is set to 0°. Each unit's preferred firing phase angle to the ripple contributes as one data point. Mean angle of the sum of all units is represented by the red arrow. The length of the red arrow is proportional to the length of the vector corresponding to the mean angle.

Field-coupled unit discharges could be identified during SPW-R as well as during carbachol-induced gamma oscillations (Fig. 2A). Spike sorting revealed 0–13 different identified units per slice in CA1 and 1–15 units in CA3 in the “*CCh*” group. Consistent with previous reports from SPW-R *in vivo*, most discharges occurred during sharp waves whereas firing outside these patterns was rare [39]. In the initial episode of SPW-R oscillations, median firing rate during SPW-R in CA1 was 0.11 Hz (P_25_: 0.03 Hz; P_75_: 0.27 Hz) compared to 0.02 Hz (0.01 Hz; 0.08 Hz) outside SPW-R (n = 100 units). Corresponding values in CA3 were 0.07 Hz (0.02 Hz; 0.16 Hz) versus 0.04 Hz (0.01 Hz; 0.09 Hz; n = 106 units). Event cross-correlations revealed that unit discharges were precisely coupled to the ∼200 Hz ripple oscillations (Fig. 2B, left and right panels; note corresponding cycle length of ∼5 ms).

Following application of carbachol, the same units that were active during previous SPW-R fired in coherence with the local gamma oscillations. Firing rates during gamma were not significantly increased when compared to the preceding SPW-R-state (Fig. 2C). Median firing rate during gamma oscillations was 0.10 Hz in CA1 (P_25_: 0.02 Hz; P_75_: 0.26 Hz) and 0.06 Hz in CA3 (P_25_: 0.02 Hz; P_75_: 0.20 Hz).

Of 100 units participating in SPW-R during the baseline recording period in CA1, 98 units also fired on SPW-R after the transient gamma period (the remaining two units fired only outside SPW-R in the second episode of analysis). Of 106 units identified in CA3, 102 participated in both SPW-R-recording episodes (two units did not fire on SPW-R after gamma, two units were recruited into SPW-R after gamma). Thus, 97% of units were stably entrained by SPW-R-oscillations. This finding reveals strong stability of participating neuronal units throughout changing network states (Fig. 2).

In experiments with ongoing SPW-R oscillations (*SPW-R*, n = 21 slices), spike sorting revealed 1–12 different identified units per slice in CA1 and 0–13 units in CA3. In CA1, median firing rate during SPW-R was 0.06 Hz (P_25_: 0.03 Hz; P_75_: 0.16 Hz) compared to 0.01 Hz (0.00 Hz; 0.06 Hz) outside SPW-R (n = 84 units). In CA3 the corresponding values were 0.05 Hz (0.02 Hz; 0.13 Hz) versus 0.03 Hz (0.01 Hz; 0.08 Hz, n = 99 units). In ongoing SPW-R recordings 98% of units fired on SPW-R in both SPW-R-recording episodes. Similarly, 100% of units recorded in the *atropine* control group stably fired on SPW-R over the course of the experiment.

For a detailed analysis of unit-to-network coupling, we restricted analysis to units firing at least 10 action potentials during each of the two SPW-R episodes (remaining neurons in the *CCh* group: n = 81 units for CA1; n = 84 for CA3, *SPW-R*: n = 66 for CA1; n = 76 for CA3, *atropine*: n = 59 for CA1, n = 65 for CA3).

### Units maintain their phase relation to ripple oscillations in CA1 after a gamma episode

As a measure for the precision of temporal coupling, we analyzed the phase angles between unit discharges and field-ripple cycles before and after the gamma episode (see Methods). Discharges showed a clear preference for firing briefly after the trough of the ripple (mean phase angle 22.2°, vector length 0.93 in CA1, n = 74 and 23.4°, 0.96 in CA3, n = 47). This phase preference was maintained after the intermittent episode of gamma oscillations (CA1: 19.9°, 0.96; CA3: 25.1°, 0.96; p>0.05 for both regions; Watson and Williams test for circular statistics; Fig. 2D). Thus, units maintained temporal coupling to SPW-R network activity after a transient period of cholinergically induced oscillations.

### Cholinergic receptor activation increases subsequent SPW-R-related firing

**Figure 3:**
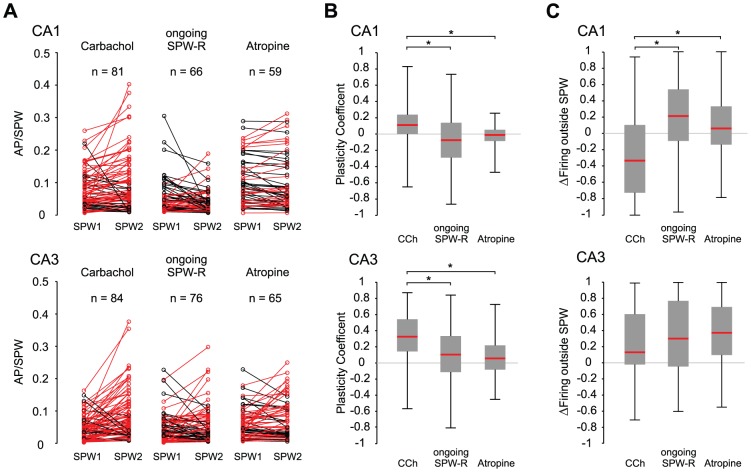
SPW-coupled firing is specifically enhanced after carbachol induced gamma oscillations in both CA1 and CA3. **A**: Each line represents one unit and the modulation of its SPW-R-related firing rate after carbachol induced gamma oscillations. Red lines illustrate potentiated units, black lines depict suppressed units. Note the dominance of enhanced units in the *CCh* experiments in contrast to ongoing SPW-R recordings. **B**: Increase of SPW-R-coupled firing (see Methods section for plasticity coefficient) was significantly stronger in the *CCh* experiments compared to ongoing SPW-R-recordings and the *atropine* control, p<0.0001 for CA1, p<0.001 for CA3; ANOVA with post-hoc test between groups). **C**: Modulation in firing outside SPW-R was calculated as a coefficient (see *c2* in Methods Section) and compared between groups. In CA1 of *CCh* experiments firing outside SPW decreased in contrast to ongoing SPW-recordings and *atropine* control (p<0.001, ANOVA and post-hoc tests). In CA3 firing outside SPWs was slightly increased in all groups. However, groups did not differ statistically (p>0.05, ANOVA).

In contrast to the constant phase relationship, intermittent carbachol-induced gamma changed the frequency of SPW-R-coupled firing. In CA1, 62 out of 81 units (76.5%) increased in firing rate whereas 19 decreased firing as compared to the first episode of SPW-R. In CA3, 70/84 units increased activity (83.3%) and 14 units decreased firing rate (Fig. 3A). In the experiments with ongoing SPW-R activity, 29/66 (43.9%) units in CA1 and 45/76 (59.2%) in CA3 increased firing in the second recording phase. In the *atropine* group 26/59 (44.1%) units in CA1 and 42/65 (64.6%) units in CA3 increased in SPW-related firing (Fig. 3A).

In order to quantify changes in SPW-R-related firing we calculated a “plasticity coefficient” (see Methods) for each unit. In CA1 units of the *CCh* group, the median plasticity coefficient was 0.12 (P_25_ 0.02; P_75_ 0.24; n = 81; Fig. 3B upper panel), indicating preferential increase in firing. Respective values for the ongoing SPW-R-oscillation group were −0.08 (P_25_ −0.29; P_75_ 0.14; n = 66), and for *atropine* controls −0.01 (P_25_ −0.08; P_75_ 0.05; n = 59). The plasticity coefficients of units from the *CCh* group were significantly different from the hypothetical mean of zero (p<0.0001, Wilcoxon signed rank test), in contrast to values from both other groups (p>0.05 for both *SPW-R* and *atropine* groups, Wilcoxon signed rank test). The changes in firing frequency observed in the *CCh* group were also significantly different from both, ongoing SPW-R-state and *atropine* control (p<0.0001; ANOVA and post-hoc tests). Data from slices with ongoing *SPW-R* and *atropine* controls were not different from each other. Firing rate of units during the initial SPW-R episode did not predict the subsequent change by transient mACh-R-induced change in network state (Spearman coefficient of correlation between both parameters: r = −0.19, p>0.05).

In CA3, wash-in of carbachol yielded an even more pronounced effect. Plasticity coefficients in the *CCh* group amounted to 0.33 (P_25_ 0.15; P_75_ 0.54, n = 84; Fig. 3B lower panel). The respective values for ongoing *SPW-R* and *atropine* control were 0.09 (P_25_ −0.12; P_75_ 0.32, n = 76) and 0.06 (P_25_ −0.08; P_75_ 0.22; Fig. 3B, n = 65). Here, measures of plasticity were different from zero (no change) in all three groups (*gamma*: p<0.0001, *SPW-R*: 0.04, *atropine*: 0.01, one-sample-t-test). However, changes in carbachol-treated slices were significantly larger than in *SPW-R* and *atropine* (p<0.001; ANOVA and post hoc tests) which were not different from each other. In addition, we found a negative correlation between initial firing rate during SPW-R and the plasticity coefficient in CA3 (r = −0.47, p<0.001), indicating that firing of units with high initial activity was less potentiated than firing of units with fewer discharges in the initial observation period. Together, these data show that unit-to-network coupling is specifically enhanced by a transient phase of cholinergic gamma oscillations.

### Firing outside SPW-R is modulated opposite to firing during SPW-R

In striking contrast to the enhanced firing during SPW-R, firing rates outside these events decreased in CA1 following application of carbachol. Conversely, firing outside SPW-R had a tendency to increase in the *SPW-R-* and *atropine*- groups. Slices undergoing transient gamma oscillations differed significantly from the other two groups in this respect (p<0.001, ANOVA and post-hoc tests, Fig. 3C). In the *CCh* group, firing rate outside SPW-R decreased and was significantly different from the hypothetical mean of zero (p<0.0001). In contrast, slices with ongoing SPW-R showed an increase in firing outside the events (p<0.05; one-sample t-test). In contrast to CA1, all three experimental groups showed increased firing outside SPW-R in CA3 (*CCh*: p<0.0001, Wilcoxon signed rank test, *SPW-R/atropine*: p<0.0001, one-sample t-test). Group values did not differ in this respect (Fig. 3C, p>0.05, ANOVA).

### Effects of carbachol-gamma on SPW-R waveforms

**Figure 4:**
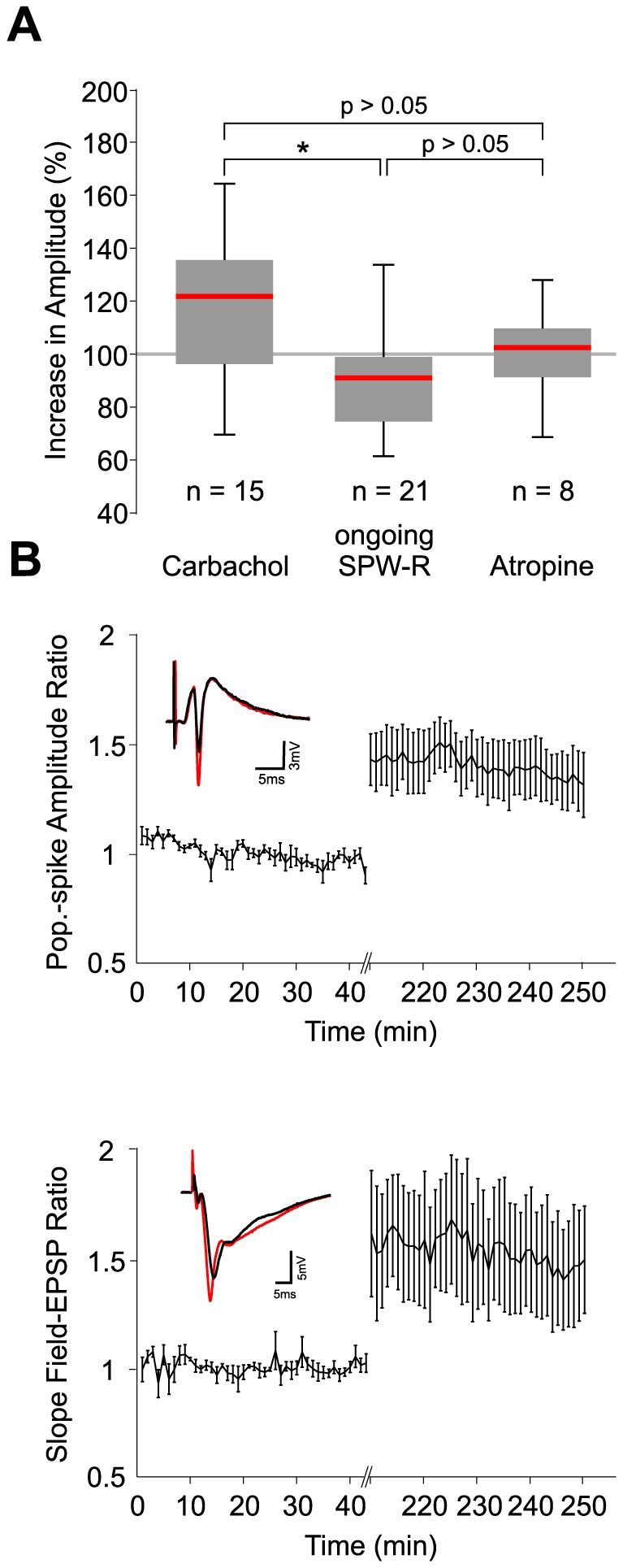
Potentiation of unit assemblies is reflected by plastic changes on the network level. **A**: Increase of amplitudes was significantly stronger after an intermittent carbachol induced gamma state than after ongoing SPW-R-oscillations. However, the increase in amplitude was not significantly different from values derived from the *atropine* control (p<0.05, *CCh* vs. *SPW-R*: p<0.01, *CCh* vs. *atropine*: p>0.05, ANOVA with post-hoc test between groups). **C**: Population spike amplitude in CA1 str. pyramidale (upper panel) and the slope of field-EPSPs in CA1 str. radiatum (lower panel) are potentiated after gamma oscillations. Intakes depict representative waveforms before (black) and after gamma (red).

SPW-R represent a complex macroscopic signal resulting from multiple cellular processes [37], [40], [41].We asked whether the observed plasticity of unit-to-network coupling was mirrored by detectable changes at the macroscopic level of field potential recordings. Sharp wave amplitude in CA1 increased following carbachol-application, reaching median amplitudes of 116%±7% compared to baseline. This value was significantly different from the ongoing *SPW-R* experiment (amplitude of late activity phase 89%±4% of initial phase) but did not differ from the *atropine* control (101%±6%, ANOVA, p>0.05 in post-hoc test; Fig. 4A). Sharp waves in CA1 comprise, at least in part, excitatory field potentials from Schaffer collaterals [26], [32], [38]. Consistent with the increase in sharp wave amplitude, the slope of evoked field-EPSPs and population spikes in stratum radiatum and pyramidale, respectively, was enhanced following application of carbachol (Fig. 4B, n = 6 slices). Thus, carbachol-induced activity potentiates spontaneous and evoked synaptic transmission between CA3 and CA1.

**Figure 5:**
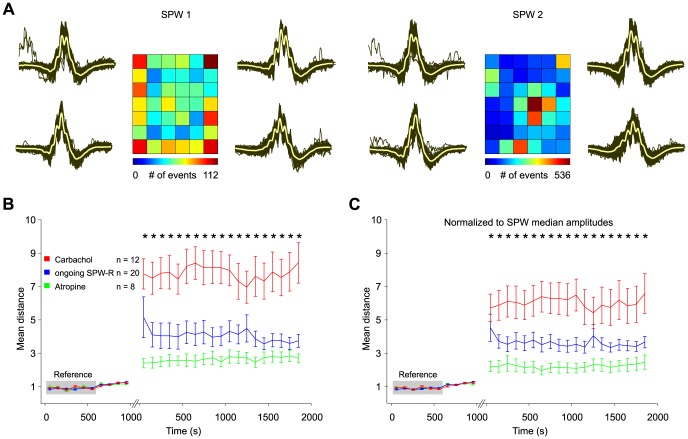
Intermittent carbachol induced gamma oscillations lead to changes in SPW-R-waveforms reflecting modulation of local neuronal assembly structure. **A**: Example of 2456 (left panel, baseline episode of 1000 s) and 7237 (right panel, post gamma episode of 2000 s) individual SPW-R events from one experiment sorted onto the respective reference SOM. Traces left and right to the SOM depict an enlarged view of the waveforms at the corner positions. Grey lines indicate individual events, yellow lines show mean waveforms which are also shown in the respective map unit. Note the shift in SPW-R-waveforms after intermittent gamma oscillations. **B**: Differences of reference maps and partial data maps after *CCh* (red), ongoing *SPW-R* oscillations (blue), and *atropine* application (green) are quantified by their mean distance to the reference episode. The shift to more different waveforms was significantly more pronounced after gamma oscillations than in the other two experimental groups (p<0.05; ANOVA with post-hoc test between groups). **C**: similar to B but baseline SPWs are normalized to the median SPW-amplitude during baseline recording and SPWs from the second recording episode are normalized to the median amplitude during that recording period. Again, gamma-induced modulation in SPW-R waveforms significantly exceeds changes in ongoing SPW-R recordings and *atropine* control (p<0.05; ANOVA with post-hoc test between groups).

We have recently shown that waveforms of individual SPW-R fall into different, transiently stable classes which reflect the composition of underlying assemblies [37]. Plastic changes of assemblies might, therefore, induce qualitative changes in SPW-R waveforms. To test this hypothesis we sorted spontaneous field events by self-organizing maps and investigated stability of waveforms over the time of each experiment (see Methods section and [37]). In all three groups, waveforms remained stable during the initial episode of SPW-R recording (Fig. 5A, 5B). Following carbachol-application, however, classification of events was clearly altered, indicating a qualitative change in waveform patterns (Fig. 5B). Similar differences between the first and the second observation period were visible in both other groups. However, changes were clearly more pronounced in slices which underwent carbachol application (Fig. 5B; p<0.05; ANOVA, Dunn's post-hoc test between groups). To eliminate a potential influence of increased SPW-R amplitude, we normalized data to this parameter (see Method section). The difference in SPW-R waveforms remained stable after this correction (Fig. 5C; p<0.05; ANOVA, Dunn's post-hoc test between groups). Thus, *CCh*-induced network activity supports plasticity at the level of single units and multi-neuronal activity patterns in hippocampal networks. In contrast, ongoing SPW-R activity is characterized by much more stable re-activation of established SPW-R waveforms and the underlying unit activity.

## Discussion

The hippocampus is involved in the formation and consolidation of memories. These distinct functions require mechanisms supporting plasticity as well as stability of neuronal activity patterns. The two-stage model of memory formation predicts that these apparently contradictory functions are bound to different states of the network. Plasticity and integration of new information happens preferentially during active wakefulness, with gamma- and theta-oscillations as the corresponding network activity [13], [42]. Consolidation of memories, on the other hand, is linked to phases of inactivity including slow-wave sleep, and is accompanied by sharp wave-ripple events [28], [29]. Stable re-play of patterns during SPW-R has been impressively demonstrated in prolonged tetrode recordings from freely moving rodents [9], [27], [43], [44].

The network state supporting plasticity is linked to strong cholinergic inputs from septal nuclei, while during SPW-R levels of acetylcholine are low [23], [24]. Indeed, acetylcholine has a well-established supportive role for synaptic plasticity in cortical networks [17], [18], [19]. However, cholinergic plasticity is not restricted to single neurons or synaptic pathways. Modern concepts of memory formation emphasize the importance of coordinated spatiotemporal activity patterns in multi-neuronal assemblies [2], [45]. Our present data constitute an *in vitro* model for cholinergic plasticity and SPW-R-related stability of neuronal assemblies. The default state of pharmacologically untreated mouse hippocampal slices is spontaneously generated sharp waves with superimposed fast (∼200 Hz) ripple oscillations [31]. Similar patterns have also been observed in rat [46], [47] and human tissue [48]. In contrast, activation of muscarinic acetylcholine receptors leads to the generation of gamma oscillations [21] which are based on an interplay between synchronously active interneurons and more sparsely firing principal cells [49], [50] Interestingly, cholinergic activity supports different forms of plasticity both at the cellular [17], [19] and at the network level [51]. These data are well compatible with the two-stage model of memory formation [11] and predict a high-cholinergic network state for memory formation and a low-cholinergic state for memory consolidation, respectively [52]. Here, we present direct evidence for the cholinergic induction of plasticity at the network level. At the same time, activity patterns during ongoing SPW-R were remarkably stable, both at the level of field potentials as well as for single units. These observations underline the stability of hippocampal assemblies during SPW-R. It is likely that the repetitive activation of existing assemblies during SPW-R [27], [43] fortifies the connections between co-active neurons. This form of plasticity would then support memory consolidation. Indeed, propagating sharp waves favor synaptic plasticity [53]. Moreover, recent evidence points towards specific cellular mechanisms of action potential generation during SPW-R which might support stabilization of coupling between single cells and the propagating network pattern [36], [54].

In our recordings, the relative timing between units and the network oscillations was remarkably stable. Similar precise timing between units and field ripples has been reported *in vivo* [39], [55] and *in vitro* [31], [32]. The underlying mechanisms are not completely clear and may include phasic synaptic inhibition [56], recurrent excitation [57] or antidromic spikes from electrically coupled axonal gap junctions [36]. In any case, the preserved phase-relationship of unit discharges during the full course of experiments indicates very stable mechanisms of coupling which are largely resistant against changes in excitatory synaptic strength.

In contrast to the stability of patterned activity during SPW-R, we observed plastic changes of unit-to network coupling during carbachol-induced state of gamma oscillations. Time-matched control recordings confirmed that the observed changes can indeed be specifically attributed to muscarinic receptor activation. Interestingly, gamma oscillations did not recruit significant numbers of new neurons into the SPW-R-related firing. The cholinergic episode did rather alter SPW-R-related neuronal firing frequency and the interplay of neurons within the assemblies during SPW-R.

Our findings thus reveal altered participation of units in the activation of multi-neuronal assemblies. Based on our previous work, we hypothesized that this plastic change in neuronal assemblies can be monitored at the field potential level [37]. Our approach was based on sorting individual SPW-R events into self-organizing maps (SOMs). This algorithm provides a classification and two-dimensional graphical illustration of the diversity and relative frequency of different SPW-R waveforms. Analysis of SPW-R waveforms during the recording time revealed stability of waveforms for control slices. In contrast, an intermittent gamma epoch caused a sudden deviation of subsequent SPW-R from initial waveforms, indicating changes in the composition or spatio-temporal activation of assemblies. Thus, plasticity of hippocampal assemblies can indeed be monitored at the near-macroscopic level of field potentials. It is likely that this activity-dependent change in field potential waveforms reflects underlying alterations in synaptic interactions and action potentials of participating cells.

Our experimental protocol was inspired by state-dependent changes in hippocampal network activity *in vivo*. We note, however, that in living animals such transitions occur rapidly and at high rates, together with changes in vigilance or behavior. SPW-R are associated with slow-wave sleep, awake immobility, and consummatory behavior whereas hippocampal gamma dominates during REM sleep and active exploratory or locomotor activity [13], [25]. We induced gamma oscillations by bath-application of cholinergic agonist carbachol which provides a pharmacological model for cholinergic input from the medial septum [21], [58]. Indeed, cholinergic tone is particularly high during active wakefulness and during REM sleep *in vivo* [52]. Hippocampal assemblies form during episodes of gamma oscillations [59], in line with the plasticity-supporting function of gamma activity [60] and cholinergic receptors [61]. In our experiments, application of carbachol induced plasticity at both the synaptic and the network level. During the gamma episode itself, i.e. under direct muscarinic influence, cellular firing rates were not markedly enhanced. This observation does largely exclude massive changes in neuronal behavior as observed during epileptiform activity in the presence of higher concentrations of carbachol [62], [63]. Additionally, in the subsequent phase of SPW-R unit firing was specifically increased during SPW-R while firing outside these events was even reduced in CA1. This points to a selective enhancement of unit firing during specific assemblies rather than an unspecific potentiation of the entire network.

It is likely that carbachol induced changes at the cellular or sub-cellular level which then translated into altered network behavior. The underlying cellular mechanisms, however, remain presently unknown, not least because the mechanisms underlying the pattern of SPW-R itself have not been completely resolved [36], [57], [64]. It is therefore difficult to predict which alterations of intrinsic neuronal properties, synaptic efficacy or electrical coupling may be responsible for the observed changes. In line with our findings, however, recent evidence shows that CA1 neurons are progressively entrained into ripple-oscillations during repetitive episodes of REM sleep [65]. Such changes of network-bound neuronal behavior may well share mechanisms with the presently observed cholinergic network plasticity. We also found excitatory transmission at Schaffer-collaterals to be potentiated after the gamma episode. This potentiation may explain the observed increase in sharp wave amplitude. These propagating positive waves certainly contain EPSPs mediated by Schaffer collateral synapses [26], [32], [38] although further mechanisms contribute to the field potential envelope [41], [66]. Further contributions may come from plastic changes at other synapses, especially recurrent excitatory connections in CA3. All these changes may preferentially affect synapses between co-active neurons, thereby contributing to the selective stabilization of pre-existing assemblies [4]. This mechanism may also explain why we did not observe integration of new, additional units to the coordinated network patterns. In principle, there were many silent neurons within reach of our tetrodes, as indicated by the large amplitude of stimulation-induced population spikes [67]. These cells were, however, not included into the strictly regulated activation of selected neurons during SPW-R which seem to support a strong separation between participating and non-participating neurons [36].

In vivo, acquisition of new memories requires integration of previously silent neurons into assemblies or silencing of previously active cells [68]. This suggests that functional states supporting learning alter the neuronal composition of hippocampal assemblies, rather than just increasing discharge frequencies of assembly-members as observed in our experiments. It is therefore likely that the interaction of living animals with their environment during active exploration activates additional mechanisms [2], [69] probably involving neuromodulatory effects of novelty detection [70], [71].

In summary, we show that a single episode of cholinergically mediated gamma oscillations induces specific changes in SPW-R, altering spatiotemporal activity patterns of assemblies and strengthening coupling between units and network. At the same time, the neuronal elements of the underlying assemblies remained remarkably constant. Cholinergic modulation of oscillating networks may provide an important link between synaptic plasticity and adaptive changes at the level of memory-associated multi-cellular networks.

## References

[1] Hebb DO (1949) The Organization of Behavior – A Neurophysiological Theory. New York, Wiley

[2] Buzsáki G (2010) Neural syntax: cell assemblies, synapsembles, and readers. Neuron 68(3): 362–85. 21040841 10.1016/j.neuron.2010.09.023 PMC3005627

[3] Buzsaki G (1989) Two-stage model of memory trace formation: a role for “noisy” brain states. Neuroscience 31: 551–570. 2687720 10.1016/0306-4522(89)90423-5

[4] Lisman JE (1999) Relating hippocampal circuitry to function: recall of memory sequences by reciprocal dentate-CA3 interactions. Neuron 22: 233–242. 10069330 10.1016/s0896-6273(00)81085-5

[5] Jensen O, Lisman JE (2005) Hippocampal sequence-encoding driven by a cortical multi-item working memory buffer. Trends Neurosci. 28(2): 67–72. 15667928 10.1016/j.tins.2004.12.001

[6] Markram H, Gerstner W, Sjöström PJ (2011) A history of spike-timing-dependent plasticity. Front Synaptic Neurosci. 3: 4. 22007168 10.3389/fnsyn.2011.00004 PMC3187646

[7] Leutgeb JK, Leutgeb S, Treves A, Meyer R, Barnes CA, et al (2005) Progressive transformation of hippocampal neuronal representations in “morphed” environments. Neuron 48: 345–358. 16242413 10.1016/j.neuron.2005.09.007

[8] Leutgeb JK, Leutgeb S, Moser MB, Moser EI (2007) Pattern separation in the dentate gyrus and CA3 of the hippocampus. Science. 315(5814): 961–6. 17303747 10.1126/science.1135801

[9] Wilson MA, McNaughton BL (1994) Reactivation of hippocampal ensemble memories during sleep. Science 265: 676–679. 8036517 10.1126/science.8036517

[10] Skaggs WE, McNaughton BL (1996) Replay of neuronal firing sequences in rat hippocampus during sleep following spatial experience. Science 271(5257): 1870–3. 8596957 10.1126/science.271.5257.1870

[11] Buzsáki G (1996) The hippocampo-neocortical dialogue. Cereb Cortex 6(2): 81–92. 8670641 10.1093/cercor/6.2.81

[12] Fell J, Axmacher N (2011) The role of phase synchronization in memory processes. Nat Rev Neurosci. 12(2): 105–18. 21248789 10.1038/nrn2979

[13] Vanderwolf CH (1969) Hippocampal electrical activity and voluntary movement in the rat. Electroencephalogr clin Neurophysiol 26: 407–418. 4183562 10.1016/0013-4694(69)90092-3

[14] Buzsáki G, Rappelsberger P, Kellényi L (1985) Depth profiles of hippocampal rhythmic slow activity (‘theta rhythm’) depend on behaviour. Electroencephalogr Clin Neurophysiol. 61(1): 77–88. 2408867 10.1016/0013-4694(85)91075-2

[15] O'Keefe J, Recce ML (1993) Phase relationship between hippocampal place units and the EEG theta rhythm. Hippocampus 3: 317–330. 8353611 10.1002/hipo.450030307

[16] Hasselmo ME, Giocomo LM (2006) Cholinergic modulation of cortical function. J Mol Neurosci. 30: 133–135. 17192659 10.1385/JMN:30:1:133

[17] Patil MM, Linster C, Lubenov E, Hasselmo ME (1998) Cholinergic agonist carbachol enables associative long-term potentiation in piriform cortex slices. J Neurophysiol. 80(5): 2467–2474. 9819256 10.1152/jn.1998.80.5.2467

[18] Ovsepian SV, Anwyl R, Rowan MJ (2004) Endogenous acetylcholine lowers the threshold for long-term potentiation induction in the CA1 area through muscarinic receptor activation: in vivo study. Eur J Neurosci. 20(5): 1267–1275. 15341598 10.1111/j.1460-9568.2004.03582.x

[19] Leung LS, Shen B, Rajakumar N, Ma J (2003) Cholinergic activity enhances hippocampal long-term potentiation in CA1 during walking in rats. J Neurosci. 23(28): 9297–9304. 14561856 10.1523/JNEUROSCI.23-28-09297.2003 PMC6740561

[20] Ovsepian SV (2006) Enhancement of the synchronized firing of CA1 pyramidal cells by medial septum preconditioning: time-dependent involvement of muscarinic cholinoceptors and GABAB receptors. Neurosci Lett. 393(1): 1–6. 16236450 10.1016/j.neulet.2005.09.035

[21] Fisahn A, Pike FG, Buhl EH, Paulsen O (1998) Cholinergic induction of network oscillations at 40 Hz in the hippocampus in vitro. Nature. 394(6689): 186–189. 9671302 10.1038/28179

[22] Kobayashi K, Poo MM (2004) Spike train timing-dependent associative modification of hippocampal CA3 recurrent synapses by mossy fibers. Neuron. 41(3): 445–54. 14766182 10.1016/s0896-6273(03)00873-0

[23] Marrosu F, Portas C, Mascia MS, Casu MA, Fà M, et al (1995) Microdialysis measurement of cortical and hippocampal acetylcholine release during sleep-wake cycle in freely moving cats. Brain Res. 671(2): 329–332. 7743225 10.1016/0006-8993(94)01399-3

[24] Monmaur P, Collet A, Puma C, Frankel-Kohn L, Sharif A (1997) Relations between acetylcholine release and electrophysiological characteristics of theta rhythm: a microdialysis study in the urethane-anesthetized rat hippocampus. Brain Res Bull. 42(2): 141–146. 8971419 10.1016/s0361-9230(96)00200-6

[25] Buzsáki G, Horváth Z, Urioste R, Hetke J, Wise K (1992) High-frequency network oscillation in the hippocampus. Science 256(5059): 1025–7. 1589772 10.1126/science.1589772

[26] Chrobak JJ, Buzsaki G (1996) High-frequency oscillations in the output networks of the hippocampal-entorhinal axis of the freely behaving rat. J Neurosci 16: 3056–3066. 8622135 10.1523/JNEUROSCI.16-09-03056.1996 PMC6579047

[27] Lee AK, Wilson MA (2002) Memory of sequential experience in the hippocampus during slow wave sleep. Neuron. 36(6): 1183–1194. 12495631 10.1016/s0896-6273(02)01096-6

[28] Girardeau G, Benchenane K, Wiener SI, Buzsáki G, Zugaro MB (2009) Selective suppression of hippocampal ripples impairs spatial memory. Nat Neurosci. 12(10): 1222–3. 19749750 10.1038/nn.2384

[29] Ego-Stengel V, Wilson MA (2010) Disruption of ripple-associated hippocampal activity during rest impairs spatial learning in the rat. Hippocampus 20(1): 1–10. 19816984 10.1002/hipo.20707 PMC2801761

[30] Jadhav SP, Kemere C, German PW, Frank LM (2012) Awake hippocampal sharp-wave ripples support spatial memory. Science. 336(6087): 1454–1458. 22555434 10.1126/science.1217230 PMC4441285

[31] Maier N, Nimmrich V, Draguhn A (2003) J Physiol 550: :873–887. Cellular and network mechanisms underlying spontaneous sharp wave-ripple complexes in mouse hippocampal slices. 10.1113/jphysiol.2003.044602 PMC2343079 12807984

[32] Both M, Bähner F, von Bohlen und HO, Draguhn A (2008) Propagation of specific network patterns through the mouse hippocampus. Hippocampus 18: 899–908. 18493949 10.1002/hipo.20446

[33] Blanche TJ, Swindale NV (2006) Nyquist interpolation improves neuron yield in multiunit recordings. J Neurosci Methods 155: 81–91. 16481043 10.1016/j.jneumeth.2005.12.031

[34] Harris KD, Henze DA, Csicsvari J, Hirase H, Buzsaki G (2000) Accuracy of tetrode spike separation as determined by simultaneous intracellular and extracellular measurements. J Neurophysiol 84: 401–414. 10899214 10.1152/jn.2000.84.1.401

[35] Hazan L, Zugaro M, Buzsaki G (2006) Klusters, NeuroScope, NDManager: a free software suite for neurophysiological data processing and visualization. J Neurosci Methods 155: 207–216. 16580733 10.1016/j.jneumeth.2006.01.017

[36] Bähner F, Weiss EK, Birke G, Maier N, Schmitz D, et al (2011) Cellular correlate of assembly formation in oscillating hippocampal networks in vitro. Proc Natl Acad Sci U S A. 108(35): E607–16. 21768381 10.1073/pnas.1103546108 PMC3167520

[37] Reichinnek S, Künsting T, Draguhn A, Both M (2010) Field potential signature of distinct multicellular activity patterns in the mouse hippocampus. J Neurosci. 30(46): 15441–9. 21084600 10.1523/JNEUROSCI.2535-10.2010 PMC6633665

[38] Csicsvari J, Hirase H, Mamiya A, Buzsaki G (2000) Ensemble patterns of hippocampal CA3-CA1 neurons during sharp wave-associated population events. Neuron 28: 585–594. 11144366 10.1016/s0896-6273(00)00135-5

[39] Csicsvari J, Hirase H, Czurko A, Mamiya A, Buzsaki G (1999) Oscillatory coupling of hippocampal pyramidal cells and interneurons in the behaving Rat. J Neurosci 19: 274–287. 9870957 10.1523/JNEUROSCI.19-01-00274.1999 PMC6782375

[40] Bedard C, Kroger H, Destexhe A (2004) Modeling extracellular field potentials and the frequency-filtering properties of extracellular space. Biophys J 86: 1829–1842. 14990509 10.1016/S0006-3495(04)74250-2 PMC1304017

[41] Lindén H, Tetzlaff T, Potjans TC, Pettersen KH, Grün S, et al (2011) Modeling the spatial reach of the LFP. Neuron 72(5): 859–72. 22153380 10.1016/j.neuron.2011.11.006

[42] Bragin A, Jandó G, Nádasdy Z, Hetke J, Wise K, et al (1995) Gamma (40–100 Hz) oscillation in the hippocampus of the behaving rat. J Neurosci. 15(1 Pt 1): 47–60. 7823151 10.1523/JNEUROSCI.15-01-00047.1995 PMC6578273

[43] Davidson TJ, Kloosterman F, Wilson MA (2009) Hippocampal replay of extended experience. Neuron. 63(4): 497–507. 19709631 10.1016/j.neuron.2009.07.027 PMC4364032

[44] Foster DJ, Wilson MA (2006) Reverse replay of behavioural sequences in hippocampal place cells during the awake state. Nature 440(7084): 680–3. 16474382 10.1038/nature04587

[45] Buzsáki G, Draguhn A (2004) Neuronal oscillations in cortical networks. Science. 304(5679): 1926–1929. 15218136 10.1126/science.1099745

[46] Kubota D, Colgin LL, Casale M, Brucher FA, Lynch G (2003) Endogenous waves in hippocampal slices. J Neurophysiol. 89(1): 81–89. 12522161 10.1152/jn.00542.2002

[47] Behrens CJ, van den Boom LP, de Hoz L, Friedman A, Heinemann U (2005) Induction of sharp wave-ripple complexes in vitro and reorganization of hippocampal networks. Nat Neurosci. 8(11): 1560–1567. 16222227 10.1038/nn1571

[48] Köhling R, Lücke A, Straub H, Speckmann EJ, Tuxhorn I, et al (1998) Spontaneous sharp waves in human neocortical slices excised from epileptic patients. Brain. 121(Pt 6): 1073–87. 9648543 10.1093/brain/121.6.1073

[49] Traub RD, Bibbig A, LeBeau FE, Buhl EH, Whittington MA (2004) Cellular mechanisms of neuronal population oscillations in the hippocampus in vitro. Annu Rev Neurosci. 27: 247–78. 15217333 10.1146/annurev.neuro.27.070203.144303

[50] Mann EO, Paulsen O (2007) Role of GABAergic inhibition in hippocampal network oscillations. Trends Neurosci. 30(7): 343–9. 17532059 10.1016/j.tins.2007.05.003

[51] Colgin LL, Kubota D, Lynch G (2003) Cholinergic plasticity in the hippocampus. Proc Natl Acad Sci U S A. 100(5): 2872–7. 12591947 10.1073/pnas.0530289100 PMC151433

[52] Hasselmo ME, McGaughy J (2004) High acetylcholine levels set circuit dynamics for attention and encoding and low acetylcholine levels set dynamics for consolidation. Prog Brain Res. 145: 207–31. 14650918 10.1016/S0079-6123(03)45015-2

[53] King C, Henze DA, Leinekugel X, Buzsáki G (1999) Hebbian modification of a hippocampal population pattern in the rat. J Physiol. 521 Pt 1: 159–167. 10562342 10.1111/j.1469-7793.1999.00159.x PMC2269637

[54] Bukalo O, Campanac E, Hoffman DA, Fields RD (2013) Synaptic plasticity by antidromic firing during hippocampal network oscillations. Proc Natl Acad Sci U S A. 26;110(13): 5175–80. 10.1073/pnas.1210735110 PMC3612622 23479613

[55] Ylinen A, Bragin A, Nadasdy Z, Jando G, Szabo I, et al (1995) Sharp wave-associated high-frequency oscillation (200 Hz) in the intact hippocampus: network and intracellular mechanisms. J Neurosci 15: 30–46. 7823136 10.1523/JNEUROSCI.15-01-00030.1995 PMC6578299

[56] Ellender TJ, Nissen W, Colgin LL, Mann EO, Paulsen O (2010) Priming of hippocampal population bursts by individual perisomatic-targeting interneurons. J Neurosci. 30(17): 5979–91. 20427657 10.1523/JNEUROSCI.3962-09.2010 PMC3763476

[57] Memmesheimer RM (2010) Quantitative prediction of intermittent high-frequency oscillations in neural networks with supralinear dendritic interactions. Proc Natl Acad Sci U S A. 107(24): 11092–7. 20511534 10.1073/pnas.0909615107 PMC2890715

[58] Traub RD, Bibbig A, Fisahn A, LeBeau FE, Whittington MA, et al (2000) A model of gamma-frequency network oscillations induced in the rat CA3 region by carbachol in vitro. Eur J Neurosci. 12(11): 4093–106. 11069606 10.1046/j.1460-9568.2000.00300.x

[59] Harris KD, Csicsvari J, Hirase H, Dragoi G, Buzsáki G (2003) Organization of cell assemblies in the hippocampus. Nature. 424(6948): 552–556. 12891358 10.1038/nature01834

[60] Magee JC, Johnston D (1997) A synaptically controlled, associative signal for Hebbian plasticity in hippocampal neurons. Science. 275(5297): 209–213. 8985013 10.1126/science.275.5297.209

[61] Hasselmo ME, Barkai E (1995) Cholinergic modulation of activity-dependent synaptic plasticity in the piriform cortex and associative memory function in a network biophysical simulation. J Neurosci. 15(10): 6592–604. 7472421 10.1523/JNEUROSCI.15-10-06592.1995 PMC6577978

[62] Egorov AV, Angelova PR, Heinemann U, Müller W (2003) Ca2+-independent muscarinic excitation of rat medial entorhinal cortex layer V neurons. Eur J Neurosci. 18(12): 3343–51. 14686907 10.1111/j.1460-9568.2003.03050.x

[63] D'Antuono M, Kawasaki H, Palmieri C, Curia G, Biagini G, et al (2007) Antiepileptic drugs and muscarinic receptor-dependent excitation in the rat subiculum. Neuropharmacology. 52(5): 1291–302. 17337018 10.1016/j.neuropharm.2007.01.008

[64] Traub RD, Schmitz D, Maier N, Whittington MA, Draguhn A (2012) Axonal properties determine somatic firing in a model of in vitro CA1 hippocampal sharp wave/ripples and persistent gamma oscillations. Eur J Neurosci. 36: 2650–2660. 22697272 10.1111/j.1460-9568.2012.08184.x PMC3433594

[65] Grosmark AD, Mizuseki K, Pastalkova E, Diba K, Buzsáki G (2012) REM Sleep Reorganizes Hippocampal Excitability. Neuron. 75: 1001–1007. 22998869 10.1016/j.neuron.2012.08.015 PMC3608095

[66] Oren I, Hájos N, Paulsen O (2010) Identification of the current generator underlying cholinergically induced gamma frequency field potential oscillations in the hippocampal CA3 region. J Physiol. 588(Pt 5): 785–97. 20051494 10.1113/jphysiol.2009.180851 PMC2834938

[67] Shoham S, O'Connor DH, Segev R (2006) How silent is the brain: is there a “dark matter” problem in neuroscience? J Comp Physiol A Neuroethol Sens Neural Behav Physiol. 192(8): 777–84. 16550391 10.1007/s00359-006-0117-6

[68] Frank LM, Brown EN, Stanley GB (2006) Hippocampal and cortical place cell plasticity: implications for episodic memory. Hippocampus 16(9): 775–84. 16921502 10.1002/hipo.20200

[69] Carr MF, Jadhav SP, Frank LM (2011) Hippocampal replay in the awake state: a potential substrate for memory consolidation and retrieval. Nat Neurosci. 14(2): 147–53. 21270783 10.1038/nn.2732 PMC3215304

[70] Lisman JE, Otmakhova NA (2001) Storage, recall, and novelty detection of sequences by the hippocampus: elaborating on the SOCRATIC model to account for normal and aberrant effects of dopamine. Hippocampus. 11(5): 551–68. 11732708 10.1002/hipo.1071

[71] Vago DR, Kesner RP (2008) Disruption of the direct perforant path input to the CA1 subregion of the dorsal hippocampus interferes with spatial working memory and novelty detection. Behav Brain Res. 189(2): 273–83. 18313770 10.1016/j.bbr.2008.01.002 PMC2421012

